# Structural Insights into a Novel Class of Aspartate Aminotransferase from *Corynebacterium glutamicum*

**DOI:** 10.1371/journal.pone.0158402

**Published:** 2016-06-29

**Authors:** Hyeoncheol Francis Son, Kyung-Jin Kim

**Affiliations:** School of Life Sciences, KNU Creative BioResearch Group, Kyungpook National University, Daehak-ro 80, Buk-ku, Daegu 702–701, Korea; University of Washington, UNITED STATES

## Abstract

Aspartate aminotransferase from *Corynebacterium glutamicum* (*Cg*AspAT) is a PLP-dependent enzyme that catalyzes the production of _L_-aspartate and α-ketoglutarate from _L_-glutamate and oxaloacetate in _L_-lysine biosynthesis. In order to understand the molecular mechanism of *Cg*AspAT and compare it with those of other aspartate aminotransferases (AspATs) from the aminotransferase class I, we determined the crystal structure of *Cg*AspAT. *Cg*AspAT functions as a dimer, and the *Cg*AspAT monomer consists of two domains, the core domain and the auxiliary domain. The PLP cofactor is found to be bound to *Cg*AspAT and stabilized through unique residues. In our current structure, a citrate molecule is bound at the active site of one molecule and mimics binding of the glutamate substrate. The residues involved in binding of the PLP cofactor and the glutamate substrate were confirmed by site-directed mutagenesis. Interestingly, compared with other AspATs from aminotransferase subgroup Ia and Ib, *Cg*AspAT exhibited unique binding sites for both cofactor and substrate; moreover, it was found to have unusual structural features in the auxiliary domain. Based on these structural differences, we propose that *Cg*AspAT does not belong to either subgroup Ia or Ib, and can be categorized into a subgroup Ic. The phylogenetic tree and RMSD analysis also indicates that *Cg*AspAT is located in an independent AspAT subgroup.

## Introduction

*Corynebacterium glutamicum* (initially reported as *Micrococcus glutamicus*) is an aerobic, gram-positive, short and rod-shaped bacterium, and is known to be involved in the fermentation of various amino acids, such as _L_-lysine and _L_-glutamate [1]. Over several decades, various *C*. *glutamicum* mutants have been isolated, which produce significant amounts of different _L_-amino acids [2]. In 2003, the genome of *C*. *glutamicum* ATCC 13032 was sequenced, providing an abundant source of data on the metabolic pathways used for the biosynthesis of industrially important products [2, 3]. During last 10 years, the market size of amino acid for livestock has been continuously increasing. _L_-methionine, _L_-lysine, _L_-threonine, _L_-tryptophan, and _L_-valine are essential amino acids manufactured industrially. [2, 4, 5].

**Fig 1:**
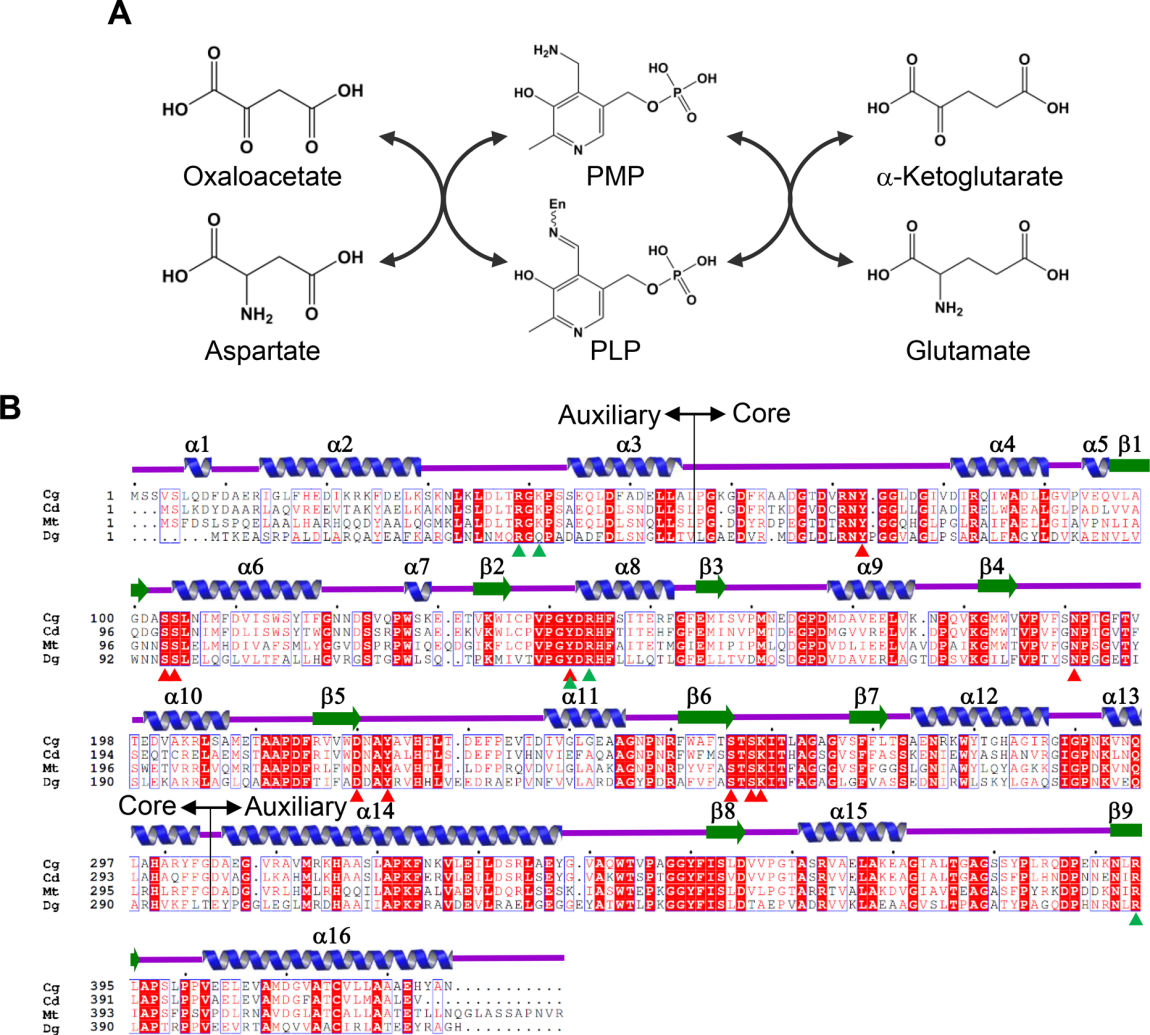
Enzyme reaction and amino acid sequence alignment of *Cg*AspAT. (A) Enzyme reaction of *Cg*AspAT. (B) Amino acid sequence alignment of AspATs from subgroup Ic. The secondary structural elements are drawn based on the structure of *Cg*AspAT. Residues involved in the binding of the PLP cofactor and the glutamate substrate are indicated by red- and green-colored triangles. The core domain and auxiliary domain are indicated as core and auxiliary, respectively. Cg, Cd, Mt and Dg are abbreviations of *Corynebacterium glutamicum*, *Corynebacterium diphtheria*, *Mycobacterium tuberculosis* and *Deinococcus geothermalis*, respectively. ‘Clustal Omega’ and ‘ESPript’ software were used for sequencing alignment.

Aspartate aminotransferase (AspAT) catalyzes the reaction from _L_-glutamate and oxaloacetate to _L_-aspartate and α-ketoglutarate [6]. AspAT utilizes pyridoxal 5’-phosphate (PLP, Vitamin B6) as a cofactor and is the most extensively studied PLP-dependent enzyme. The catalysis of AspAT consists of two reaction steps. In the first reaction step, the enzyme converts amino acid substrate to ketoacid while the PLP bound in the enzyme (internal aldimine form) is converted to pyridoxamine phosphate (PMP). In the second reaction step, another ketoacid is converted to the amino acid product, and PMP returns to its initial state (Fig 1A) [7, 8].

Aminotransferases (ATs) have been classified into five classes by evolutionary relationship. Class I includes aspartate AT, phenylalanine AT, and tyrosine AT, and class II contains glycine AT, arginine AT, and lysine AT. And, class III contains ornithine AT, isoleucine AT, leucine AT, valine AT and _D_-amino acid AT, and class IV contains phosphoserine AT and serine AT. Finally, class V contains serine-pyruvate AT [9–11]. Among the five classes, AspAT proteins belong to class I AT that is further divided into 2 subcategories, subgroup Ia and Ib [9, 12]. Most eukaryotic AspAT enzymes, such as *Gallus gallus*, *Sus scrofa*, *Saccharomyces cerevisiae*, *Plasmodium falciparum*, *Gaiardia lamblia*, *Trypanosoma brucei*, and *Leishmania major* and several bacterial AspAT enzymes including *Escherichia coli* were classified into subgroup Ia, whereas other bacterial AspAT enzymes, such as *Thermus thermophilus* and *Thermotoga maritime*, were classified into subgroup Ib [12–21].

In this study, we report a crystal structure of AspAT from *Corynebacterium glutamicum* ATCC13032 (*Cg*AspAT) in complex with a PLP and citrate molecule. Compared with AspATs from subgroup Ia and Ib, *Cg*AspAT showed unusual structural features at the auxiliary domain and unique binding sites for both cofactor and substrate. Based on these observations, we propose that *Cg*AspAT can be categorized into a new subgroup, subgroup Ic.

## Materials and Methods

### Cloning, expression and purification

The gene coding *Cg*AspAT was amplified through polymerase chain reaction (PCR) using *C*. *glutamicum* strain ATCC 13032 chromosomal DNA as the template. The PCR products were sub-cloned into pET30a (Novagen), and the resulting expression vector pET30a:*Cg*AspAT was transformed into a *E*. *coli* BL21(DE3)-T1^R^ strain, which was grown in 1 L of LB medium containing 100 μM kanamycin at 310 K. At an OD_600_ of 0.65, *Cg*AspAT protein expression was induced by adding 1 mM IPTG. After 20 h at 293 K, the cells were harvested through centrifugation at 4,000 *g* for 20 min at 277 K. The cell pellet was resuspended in buffer A (40 mM Tris-HCl pH 8.0) and disrupted by ultrasonication. The cell debris was removed through centrifugation at 13,500 *g* for 25 min, and the lysate was applied onto a Ni-NTA agarose column (Qiagen). After washing with buffer B (40 mM Tris-HCl pH 8.0 and 30 mM imidazole), the bound proteins were eluted with buffer C (40mM Tris-HCl pH 8.0 and 300 mM imidazole). Finally, the trace amount of contaminants was removed by size-exclusive chromatography using a Sephacryl S-300 prep-grade column (320 ml, GE Healthcare) equilibrated with buffer A. The eluted protein had a molecular weight of about 90 kDa, indicating a dimeric structure. The protein was concentrated to 50 mg mL^-1^ using spin column (Amicon Ultra Centrifugal Filter, 30 kDa pore size), and kept at 193 K for further experiments. All purification steps were performed at 277 K.

### Crystallization and data collection

Crystallization of the purified *Cg*AspAT protein was initially performed with commercially available sparse-matrix screens including Index, PEG I and II (from Hampton Research), Wizard Classic I and II, Wizard CRYO I and II (from Rigaku Reagents), and Structure Screen I and II (from Molecular Dimensions), using the sitting-drop vapor-diffusion method on the MRC Crystallization plate (Molecular Dimensions) at 295 K. Each experiment consisted of mixing 1.0 μL protein solution (50 mg·mL^−1^, 40 mM Tris-HCl pH 8.0) with 1.0 μL reservoir solution and then equilibrating against 50 μL reservoir solution. *Cg*AspAT crystals were observed in several crystallization screening conditions. After several steps of improvement using the hanging-drop vapor-diffusion methods, crystals of the best quality appeared using 28% polyethylene glycol 3350 and 200 mM Ammonium citrate dibasic as reservoir solution and protein concentration of 50 mg·mL^−1^, were finished out with a loop larger than the crystals and flash-frozen by immersion in liquid nitrogen at 100 K. The data were collected to a resolution of 2.0 Å at beamline 7A at the Pohang Accelerator Laboratory (PAL, Pohang, Republic of Korea) using a Quantum 270 CCD detector (ADSC, USA) (Table 1). All data was indexed, integrated, and scaled together using the HKL2000 software package [22]. The crystals of *Cg*AspAT belonged to the space group C2. Assuming two *Cg*AspAT molecules in the asymmetric unit, the crystal volume per unit of protein mass was 2.45 Å^3^·Da^−1^, which means that the solvent content was 49.92% [23].

**Table 1:** Data collection and refinement statistics.

	*Cg*AspAT
**Data collection**	
Space group	C2
Wavelength (Å)	0.97934
Cell dimensions	
*a*, *b*, *c* (Å)	103.4, 53.5, 170.2
α, β, γ (°)	90.00, 104.23, 90.00
Resolution (Å)	50.00–2.00 (2.03–2.00)*
*R*_sym_ or *R*_merge_	4.9 (9.0)*
*I* / σ (*I*)	41.62 (25.43)*
Completeness (%)	97.7 (98.2)*
Redundancy	3.6 (3.7)*
**Refinement**	
Resolution (Å)	50.00–2.00
No. reflections	56893
*R*_work_ / *R*_free_	14.8 / 19.9
No. atoms	7452
Protein	6591
Ligand/ion	105
Water	756
*B*-factors	14.5
Protein	13.6
Ligand/ion	33.6
Water	25.4
R.m.s. deviations	
Bond lengths (Å)	0.0191
Bond angles (°)	1.8846
Ramachandran statistics (%)	
Favored	96.0
Allowed	3.4
Outliers	0.6

*Values in parentheses are for highest-resolution shell.

### Structure determination

The structure was determined by molecular replacement using the CCP4 version of MOLREP [24] and the structure of the putative aminotransferase from *Corynebacterium diphtheria* (PDB code 3D6K) as the search model. Model building was performed manually using the program WinCoot [25], and refinement was performed with CCP4 refmac5 [26] and CNS [27]. The data statistics are summarized in Table 1. The refined *Cg*AspAT model was deposited in protein data bank (PDB code 5IWQ).

### Site-directed mutagenesis and activity assay

Site-specific mutations were created using the Quick Change kit (Stratagene), and sequencing was performed to confirm the correct incorporation of the mutations. Mutant proteins were purified in the same manner as the wild type. Because the reaction of AspAT cannot detect directly, the *Cg*AspAT activity assay was performed in duplicate reaction with malate dehydrogenase. At the first reaction, *Cg*AspAT enzymes catalyze _L_-asparate and α-ketoglutarate to _L_-glutamate and oxaloacetate, and at the second reaction, oxaloacetate is converted to malate, by malate dehydrogenase using NADH as a cofactor. Oxidation of NADH to NAD was monitored through the decrease of absorbance at 340 nm (extinction coefficient of 6.22 x 10^3^ M^−1^·cm^−1^) (S1 Fig). All assays were formed with a reaction mixture of 1 mL total volume, and performed at 303 K. The reaction mixture contained 100 mM Tris-HCl, pH 8.0, 300 mM _L_-aspartate, 50 mM α-ketoglutarate, 80 μM NADH, and 10 unit·mL^−1^ malate dehydrogenase. The reaction was initiated by the addition of enzyme to a final concentration of 1 μg·mL^−1^. The decreased absorbances at 340 nm for 20 sec after the initiation of the reaction were measured.

## Results and Discussion

### Overall structure of *Cg*AspAT

In order to elucidate the molecular mechanism of *Cg*AspAT, we determined the crystal structure of the protein at 2.0 Å. There were two *Cg*AspAT molecules in each asymmetric unit, which form a homodimer of the protein. The atomic structure was in good agreement with the X-ray crystallographic statistics for bond angles, bond lengths, and other geometric parameters (Table 1).

**Fig 2:**
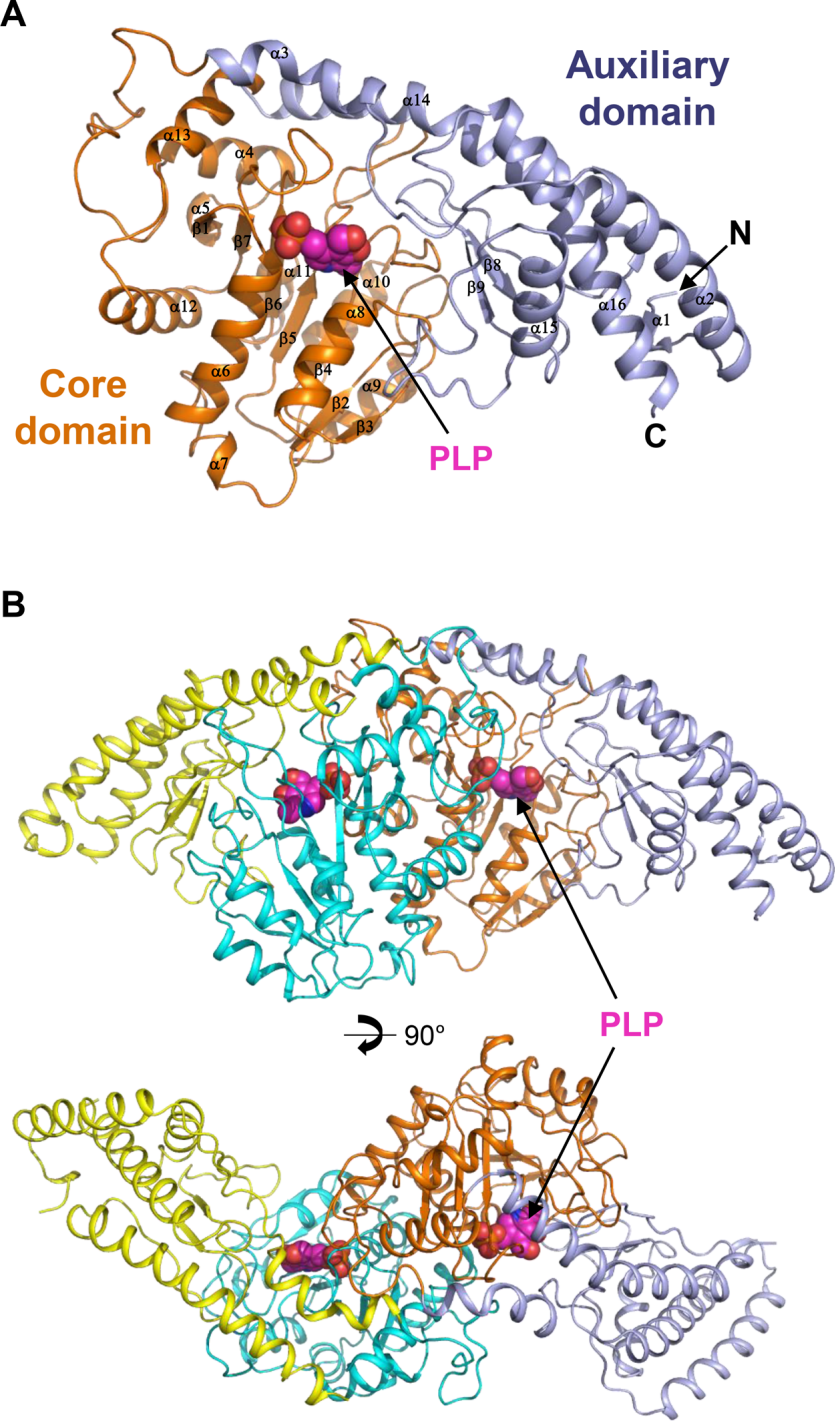
Overall structure of *Cg*AspAT. (A) The monomeric structure of *Cg*AspAT. The monomeric structure of *Cg*AspAT is presented as a cartoon diagram. The core domain and the auxiliary domain are distinguished with orange and light-blue colors, respectively, and labeled. The bound PLP cofactor is shown as a magenta-colored sphere model. Secondary structure elements are labeled. (B) Dimeric structure of *Cg*AspAT. The dimeric structure of *Cg*AspAT is presented as a cartoon diagram. The bound PLP cofactor is shown as a magenta-colored sphere model. The bottom figure is rotated by 90 degree from the top.

The monomer structure of *Cg*AspAT consists of two domains: a core domain (Leu56-Gly304) and an auxiliary domain (Ala55-Lys34 and Asp305-Asn426). The core domain has 10 α-helices (α4-α13) that surround the 7-stranded (β1-β7) β-sheet located in the middle of the domain (Figs 1B and 2A). The auxiliary domain has 6 α-helices (α1- α3 and α14-α16), with five arranged in a line, and 2 β-strands (β8-β9), which are located between the α-helix line and the core domain (Figs 1B and 2A). Dimerization is mainly mediated through the contact between the core domains of the two subunits with three α-helices (α6, α8, and α12) of one molecule in contact with the corresponding α-helices of the other molecule (Fig 2B). For dimerization, 63 amino acids are involved in each monomer, and a total of 2472.5 and 2486.2 Å^2^ of solvent-accessible surface areas per each monomer are buried, which correspond to 13.9% of the total surface areas of each monomer, respectively. The cofactor- and substrate-binding pockets are at the domain interface, which will be described in the following sub-sections.

## PLP binding mode and catalytic residue of *Cg*AspAT

**Fig 3:**
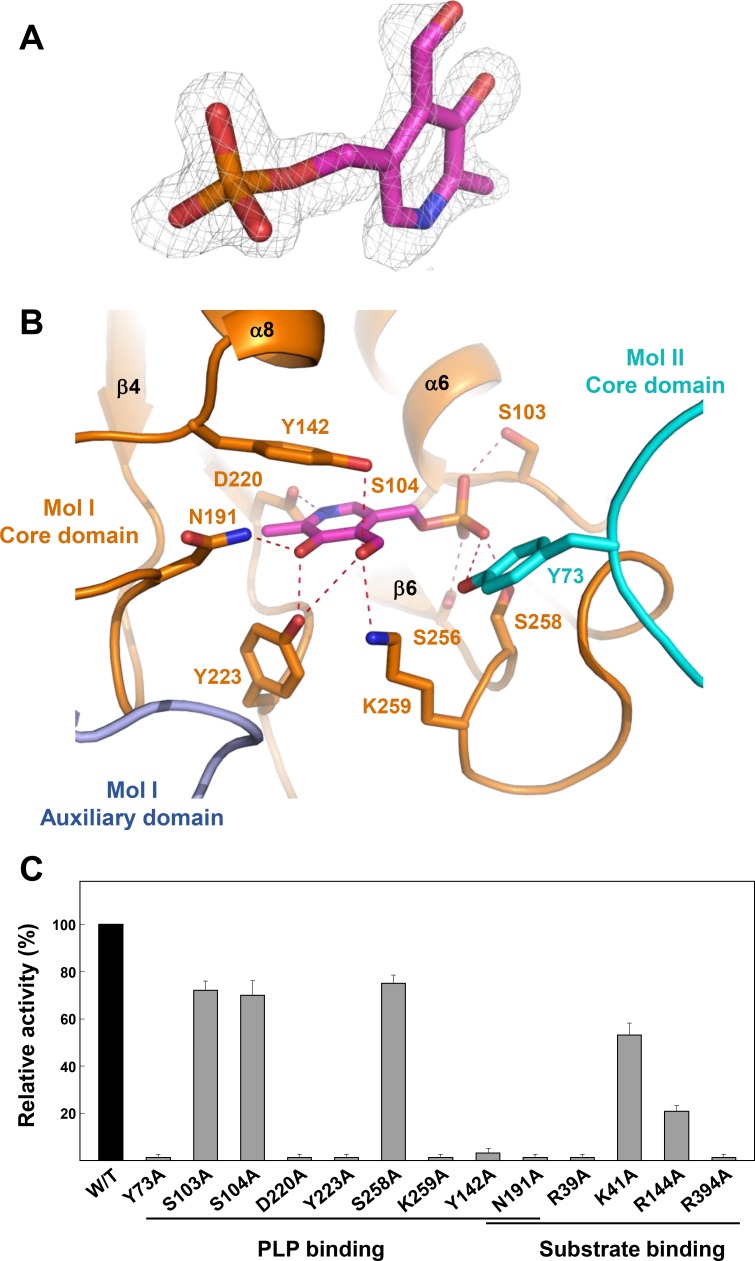
PLP binding mode of *Cg*AspAT. (A) Electron density map of the bound PLP. The 2Fo-Fc electron density map of the bound PLP in *Cg*AspAT is shown with a gray-colored mesh, and contoured at 1.2 σ. (B) PLP binding mode of *Cg*AspAT. The bound PLP cofactor is presented as a stick model with a magenta color. Residues involved in the binding of PLP are shown as stick models and labeled appropriately. The core domain and the auxiliary domain of one molecule are distinguished with orange and light-blue colors, respectively, and the other molecule is with a cyan color. Hydrogen bonds involved in the PLP binding are shown with red-colored dotted lines. (C) Site-directed mutagenesis of *Cg*AspAT. Residues involved in binding of cofactor and substrate are replaced by alanine residues. The relative activities of recombinant mutant proteins were measured and compared with that of wild-type *Cg*AspAT.

Since we observed the electron density for the PLP cofactor in our structure without adding it in the crystallization process (Fig 3A), we speculate that the PLP cofactor binds strongly to *Cg*AspAT. Moreover, the enzyme exhibited AspAT activity without the addition of PLP in the reaction mixture, supporting the high affinity between the enzyme and the PLP cofactor. The PLP-binding pocket is composed of six loops (α3-α4, β1-α6, β2-α8, β4-α10, β5-α11, and β6-β7). Pyridoxal ring was stabilized by the hydrogen bonds with residues Tyr142, Asn191, Asp220, Tyr223, and Lys259. The nitrogen atom of pyridine ring was hydrogen bonded with Asp220, and the carbonyl group with Asp220 and Tyr223. Tyr223 was also involved in the interaction with the aldehyde-group with the aid of Tyr142 and Lys259 (Fig 3B). Conserved hydrophobic residues, such as Leu105 and Val186, also contribute to the stabilization of pyridoxal ring, among the residues involved in PLP binding, Asn191, Asp220, and Tyr223 were conserved in AspAT enzymes from other organisms (Figs 1B and 3B).

Interestingly, the phosphate moiety was stabilized in a unique mode. Unlike the binding of the phosphate moiety in other AspAT enzymes, where side chains of arginine, threonine, and one or two serine residues are involved, the moiety of *Cg*AspAT was stabilized by the side chains of one tyrosine (Tyr73) and four serine residues (Ser103, Ser104, Ser256, and Ser258) through a hydrogen bond network (Fig 3B). In detail, Tyr73 and Ser258 form hydrogen bonds with the O1 atom, and Ser103 and Ser256 stabilize the O2 and O3 atoms, respectively. Moreover, Ser104 forms hydrogen bonds with both O3 and O4 atoms (Fig 3B). Near the aldehyde group of the bound PLP, we observed the conserved Lys259 residue. Since this lysine residue is known to function as a catalytic residue in other AspAT enzymes by forming an enzyme aldimine covalent bond, we speculate that *Cg*AspAT may catalyze the enzyme reaction in a way similar to other AspAT enzymes. The residues involved in the cofactor binding were further confirmed by site-directed mutagenesis experiments. Mutants, such as Y73A, Y142A, N191A, D220A, Y223A, and K259A, exhibited almost complete loss of enzyme activity, indicating that these residues are crucial for PLP binding. However, mutations of serine residues (S103A, S104A, and S258A) involved in the stabilization of the phosphate moiety showed ~70% AspAT activities compared with the wild type, indicating that these residues, individually, do not influence much the stabilization of the phosphate moiety (Fig 3C).

### Substrate binding mode of *Cg*AspAT

**Fig 4:**
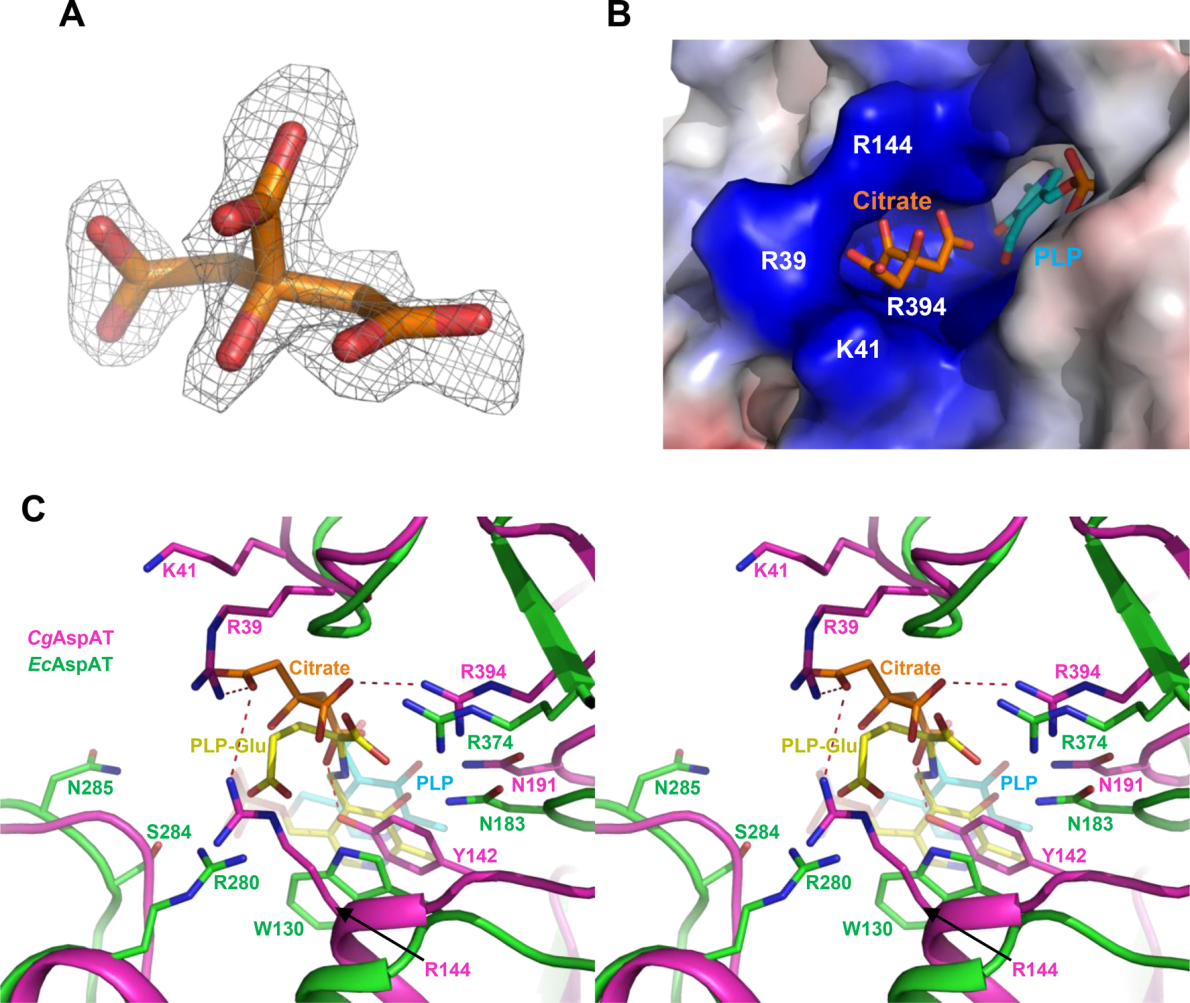
Substrate binding mode of *Cg*AspAT. (A) Electron density map of the bound citrate in *Cg*AspAT. The electron density maps Fo-Fc of the bound citrate in *Cg*AspAT is shown with a gray-colored mesh, and contoured at 2.5 σ (B) Electrostatic potential surface model of the substrate binding site. The *Cg*AspAT structure is presented as an electrostatic potential surface model. The bound PLP and citrate are shown as stick models with cyan and orange colors, respectively. The highly positively-charged residues constituting the substrate binding site are labeled. (C) Stereo-view of substrate binding mode of *Cg*AspAT. The *Cg*AspAT structure is superposed with the *Ec*AspAT structure in complex with PLP-glutamate. Structures of *Cg*AspAT and *Ec*AspAT are shown as cartoon diagram with magenta and green colors, respectively. Residues involved in the glutamate binding are shown as a stick model and labeled appropriately. The PLP-glutamate bound in *Ec*AspAT is shown as a stick model with a yellow color, and the PLP molecule and the citrate ion bound in *Cg*AspAT are with colors of cyan and orange, respectively.

In our current structure, one citrate molecule, a component of the crystallization solution, is observed to be bound (Fig 4A and 4B), and the superposition of our structure with the AspAT from *Escherichia coli* (*Ec*AspAT) structure in complex with the glutamate substrate (pdb code 1X28) allows us to speculate the substrate binding of *Cg*AspAT. In *Ec*AspAT, the α-carboxyl group of glutamate is stabilized by hydrogen bonds with Asn183 and Arg374 residues. The α-carboxyl group of glutamate might be similarly stabilized in *Cg*AspAT, because residues of Asn191 and Arg394 are located at the corresponding positions of Asn183 and Arg374, respectively (Fig 4C). However, the binding mode of the γ-carboxyl group of glutamate in *Cg*AspAT seems to be quite different compared with that in *Ec*AspAT. In *Ec*AspAT, four residues such as Trp130, Arg280, Ser284, and Asn285 are involved in stabilization of the γ-carboxyl group of glutamate by direct and water-mediated hydrogen bonds, and none of these residues are conserved in *Cg*AspAT. In *Cg*AspAT, Arg39, Tyr142 and Arg144 are positioned in the vicinity of the γ-carboxyl group of glutamate (Fig 4C). As observed above, in *Cg*AspAT, a citrate molecule is bound at the position that the glutamate substrate is bound in *Ec*AspAT. Because the citrate molecule is stabilized by hydrogen bonds with the highly positively-charged residues such as Arg39, Lys41, Arg144 and Arg394, binding of the citrate molecule seems to mimic the substrate binding of *Cg*AspAT (Fig 4B and 4C). The involvement of these residues in substrate binding is further confirmed by site-directed mutagenesis experiments. When we replace the Arg39, Tyr142, Arg144, Asn191 and Arg394 residues by alanine, all mutants showed reduced or almost complete loss of enzyme activity, indicating that these unique residues are crucial for the binding of the glutamate substrate in *Cg*AspAT (Fig 3C).

### Comparison of *Cg*AspAT with subgroup Ia and Ib

AspAT enzymes belong to the aminotransferase class I. Based on amino acid sequence similarity, class I was further divided into two subgroups, subgroup Ia and Ib. Although amino acid sequence similarities within enzymes in subgroup Ia and Ib are about 40%, that between enzymes in subgroups Ia and Ib is about 15% [12]. Subgroup Ia includes mostly eukaryotic and some bacterial AspAT enzymes, while some extremophiles belong to subgroup Ib. However, we observed that *Cg*AspAT has unique structural features compared with AspAT enzymes from subgroups Ia and Ib, and proposed that *Cg*AspAT can be categorized as a new subgroup due to the following reasons.

**Fig 5:**
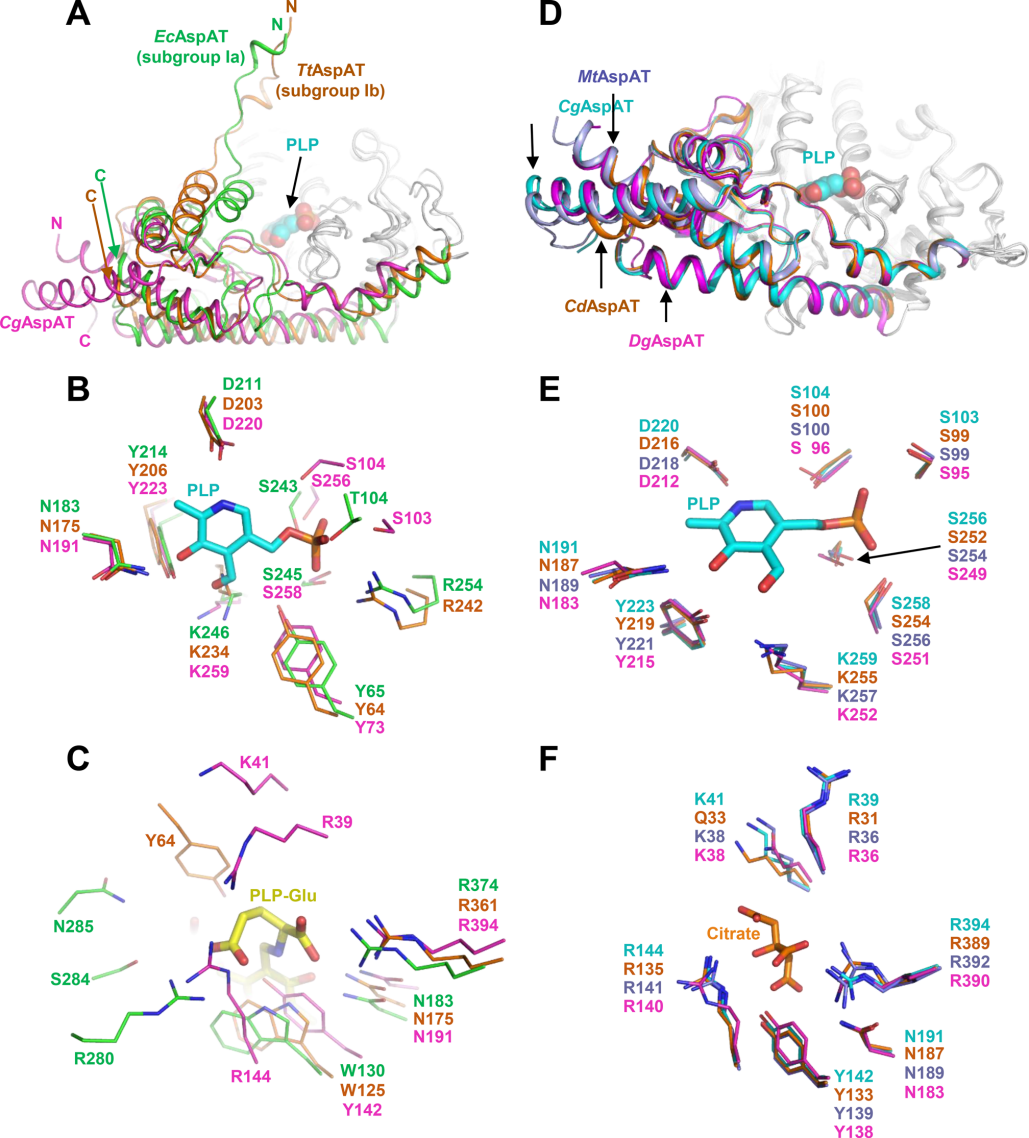
Comparison of *Cg*AspAT with subgroup Ia and Ib. (A) Comparison of the auxiliary domain of *Cg*AspAT with those of AspATs from *E*. *coli* (*Ec*AspAT, subgroup Ia) and *T*. *thermophilus* (*Tt*AspAT, subgrpup Ib). Structures of *Ec*AspAT, *Tt*AspAT and *Cg*AspAT are superimposed. The core domains of these enzymes are shown with a gray color, and the auxiliary domains of *Ec*AspAT, *Tt*AspAT and *Cg*AspAT are distinguished with green, orange and magenta colors, respectively. The bound PLP and glutamate in *Cg*AspAT are shown as sphere model with magenta and cyan colors, respectively. RMSD values for *Ec*AspAT-*Tt*AspAT, *Ec*AspAT-*Cg*AspAT, and *Tt*AspAT-*Cg*AspAT are 2.73, 3.39, and 2.93, respectively. *Ec*AspAT, *Tt*AspAT and *Cg*AspAT are representative AspATs for subgroup Ia, Ib and Ic, respectively. (B) Comparison of the cofactor binding mode of *Ec*AspAT, *Tt*AspAT and *Cg*AspAT. Structures of *Ec*AspAT, *Tt*AspAT and *Cg*AspAT are superimposed. Residues involved in the PLP cofactor binding are shown as a stick model with green, orange and magenta colors for *Ec*AspAT, *Tt*AspAT and *Cg*AspAT, respectively. The bound PLP in *Cg*AspAT is shown as a stick model with a cyan color. (C) Comparison of the substrate binding mode of *Ec*AspAT, *Tt*AspAT and *Cg*AspAT. Structures of *Ec*AspAT, *Tt*AspAT and *Cg*AspAT are superimposed. Residues involved in the substrate binding are shown as a stick model with green, orange and magenta colors for *Ec*AspAT, *Tt*AspAT and *Cg*AspAT, respectively. The bound PLP-glutamate in *Ec*AspAT is shown as a stick model with a yellow color. (D) Comparison of the auxiliary domain of AspATs from subgroup Ic. Structures of *Cg*AspAT, *Cd*AspAT, *Mt*AspAT and *Dg*AspAT are superimposed. The core domains of these enzymes are shown with a gray color, and the auxiliary domains of *Cg*AspAT, *Cd*AspAT, *Mt*AspAT and *Dg*AspAT are distinguished with cyan, orange, light blue and magenta colors, respectively. The bound PLP and glutamate in *Cg*AspAT are shown as stick models with magenta and cyan colors, respectively. RMSD values for *Cg*AspAT-*Cd*AspAT, *Cg*AspAT-*Mt*AspAT, *Cg*AspAT-*Dg*AspAT, *Cd*AspAT-*Mt*AspAT, *Cd*AspAT-*Dg*AspAT, and *Mt*AspAT-*Dg*AspAT are 0.65, 0.12, 1.33, 0.66, 1.38, and 1.35, respectively. (E) Comparison of the cofactor binding mode of AspATs from subgroup Ic. Structures of *Cg*AspAT, *Cd*AspAT, *Mt*AspAT and *Dg*AspAT are superimposed. Residues involved in the PLP cofactor binding are shown as a stick model with cyan, orange, light blue and magenta colors for *Cg*AspAT, *Cd*AspAT, *Mt*AspAT and *Dg*AspAT, respectively. The bound PLP in *Cg*AspAT is shown as a stick model with a cyan color. (F) Comparison of the substrate binding mode of AspATs from subgroup Ic. Structures of *Cg*AspAT, *Cd*AspAT, *Mt*AspAT and *Dg*AspAT are superimposed. Residues involved in the substrate binding are shown as a stick model with cyan, orange, light blue and magenta colors for *Cg*AspAT, *Cd*AspAT, *Mt*AspAT and *Dg*AspAT, respectively. The bound citrate in *Cg*AspAT is shown as a stick model with an orange color.

First, although the overall fold of the core domain in the case of *Cg*AspAT is similar to that of other AspAT enzymes from subgroup Ia and Ib, the auxiliary domain of *Cg*AspAT showed a unique structural conformation. In AspATs from subgroups Ia and Ib, the N-terminal region is positioned in the vicinity of the core domain, constituting part of the substrate binding site. However, in *Cg*AspAT, the N-terminal region is positioned away from the core domain and is not involved in the formation of the substrate binding-site (Fig 5A).

Second, *Cg*AspAT uses unique residues for the stabilization of the PLP cofactor. Although most AspATs share conserved residues for enzyme catalysis and stabilization of the pyridoxal ring binding, the binding mode of the phosphate moiety was quite different from the other subgroups. In AspATs from subgroup Ia, one each of Thr, Tyr, and Arg and two Ser residues are involved in binding phosphate moiety, while in subgroup Ib only one each of Ser, Tyr and Arg residues. However, *Cg*AspAT uses four Ser and one Tyr residues for the stabilization of the phosphate moiety (Fig 5B). Moreover, two hydrophobic residues, Leu105 and Val186 that contribute to the stabilization of PLP in *Cg*AspAT, are not conserved in enzymes in subgroup Ia and Ib. In the position of Leu105, Lys and Thr are positioned in subgroup Ia and Ib, respectively. And in the position of Val186, His and Asn are positioned in subgroup Ia and Ib, respectively. These residues are quite conserved within their subgroups, indicating that AspATs from different subgroups have distinctive PLP binding modes (Fig 1B, S2 and S3 Figs).

Finally, *Cg*AspAT also has a unique mode for substrate binding. In AspATs from subgroup Ia, residues, such as two Arg, Asn, and Trp (or Tyr), are involved in substrate binding. AspATs from subgroup Ib stabilize the substrate in a way similar to those from AspATs from subgroup Ia, where Arg, Asn, Tyr, and Trp (or Tyr) are involved. On the other hand, *Cg*AspAT utilizes unique residues, such as one each of Tyr and Lys, dyad and three Arg residues (Fig 5C). As observed in the residues involved in the PLP binding, these residues are also highly conserved within its subgroup, and we suspect that these unique substrate binding-modes may be one of the decisive structural features of each subgroup (Fig 1B, S2 and S3 Figs). Interestingly, we found three unpublished AspAT structures in the protein data bank, AspATs from *Corynebacterium diphtheria* (*Cd*AspAT, PDB Code: 3D6K), *Mycobacterium tuberculosis* (*Mt*AspAT, PDB Code: 5C6U) and *Deinococcus geothermalis* (*Dg*AspAT, PDB Code: 3EZ1), and these enzymes have structural features similar to *Cg*AspAT (Fig 5D, 5E and 5F), indicating that these enzymes can belong to the category same as *Cg*AspAT.

**Fig 6:**
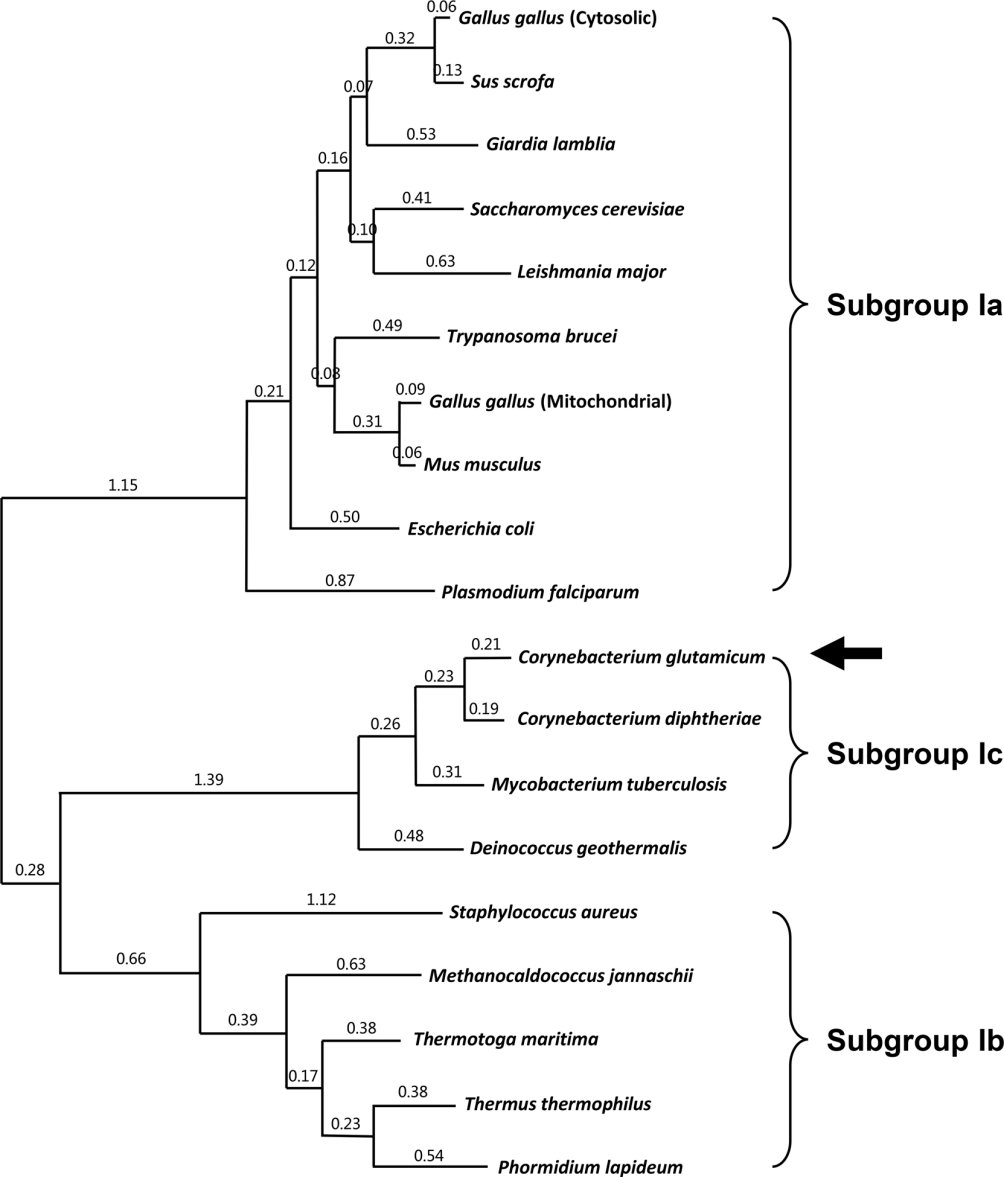
Phylogenetic relationships between AspAT enzymes. The maximum likelihood tree was calculated using MEGA (Molecular Evolutionary Genetics Analysis) software on the basis of a Clustal Omega. Alignment of AspAT enzymes from three different subgroups. Ten, five, and four AspAT enzymes are selected for subgroup Ia, Ib, and Ic, respectively.

We then performed a phylogenetic tree analysis of AspATs from various organisms (Fig 6). As expected, AspATs from subgroup Ia and subgroup Ib were located away from each other. However, *Cg*AspAT did not belong to either subgroup Ia or subgroup Ib, confirming that *Cg*AspAT can be categorized as a new subgroup. Moreover, *Cd*AspAT, *Mt*AspAT and *Dg*AspAT enzymes show ~40% amino acid sequence similarity with *Cg*AspAT and are located close to *Cg*AspAT in the phylogenetic analysis (Fig 6). Here, combined with the unique structural features described above, we propose that *Cg*AspAT, together with *Cd*AspAT, *Mt*AspAT and *Dg*AspAT enzymes, can be categorized as subgroup Ic.

## Conclusion

We determined the crystal structure of *Cg*AspAT in complex with the PLP cofactor and we speculated glutamate substrate binding site compare to *Ec*AspAT structure. Many biochemical and structural studies on AspATs have been reported for decades, which categorize these enzymes into two subgroups, subgroup Ia and subgroup Ib. However, *Cg*AspAT shows only 10~15% amino acid sequence similarity with AspATs from subgroup Ia and subgroup Ib and exhibits unique structural features not only in the auxiliary domain but also at the catalytic and substrate binding sites. These amino acid sequence analysis and the structural comparisons reveal that *Cg*AspAT does not belong to either subgroup Ia or subgroup Ib, but rather should be classified to a new category, subgroup Ic. Moreover, the RMSD analysis of the reported AspAT structures supports new classification of enzymes in this family (S1 Table.).

## Supporting Information

S1 Fig Two step reaction used for measurement of aspartate aminotransferase activity. Coupled reaction is used to measure aminotransferase activity. At the 1^st^ reaction and 2^nd^ reaction, aspartate aminotransferase (AspAT) and malate dehydrogenase (MDH) are used respectively, and the decrease amount of NADH is monitored at 340nm.
(TIF)

S2 Fig Amino acid sequence alignment of AspAT enzymes from subgroup Ia. Amino acid sequences of 10 representative AspAT enzymes from subgroup Ia are aligned using Clustal Omega and ESPript software. The secondary structure elements are shown based on the structure of AspAT from *E*. *coli*. Residues involved in the binding of the PLP cofactor and the glutamate substrate are indicated by red- and green-colored triangles.
(TIF)

S3 Fig Amino acid sequence alignment of AspAT enzymes from subgroup Ib. Amino acid sequences of 5 representative AspAT enzymes from subgroup Ib are aligned using Clustal Omega and ESPript software. The secondary structure elements are shown based on the structure of AspAT from *Thermus thermophilus*. Residues involved in the binding of the PLP cofactor and the glutamate substrate are indicated by red- and green-colored triangles.
(TIF)

S1 Table RMSD analysis of reported AspAT structures. (A) RMSD of reported AspAT structures were analyzed. PDB code 1SPA; *Escherichia coli*, 7AAT; *Gallus gallus* (cytosolic), 2CST; *Gallus gallus* (mitochondrial), 3PD6; *Mus musculus*, 4W5K; *Trypanosoma brucei*, 1AJR; *Sus scrofa*, 3MEB; *Gaiardia lamblia*, 1YAA; *Saccharomyces cerevisiae*, 4H51; *Leishmania major*, 3K7Y; *Plasmodium falciparum*, 1B5O; *Thermus thermophilus*, 1O4S; *Thermotoga maritima*, 1J32; *Phormidium lapideum*, 2Z61; *Methanocaldococcus jannaschii*, 2O1B; *Staphylococcus aureus*, Cg; *Corynebacterium glutamicum*, 3D6K; *Corynebacterium diphtheriae*, 5C6U; *Mycobacterium tuberculosis*, 3EZ1; *Deinococcus geothermalis*. (B) Average RMSD values between AspAT structures of 3 different subgroups.
(TIF)
