# Supplementary figures and images for: Structural Insights into a Novel Class of Aspartate Aminotransferase from Corynebacterium glutamicum
Source: PLoS One. 2016 Jun 29;11(6):e0158402. doi: 10.1371/journal.pone.0158402 (PMC4927141; doi:10.1371/journal.pone.0158402)

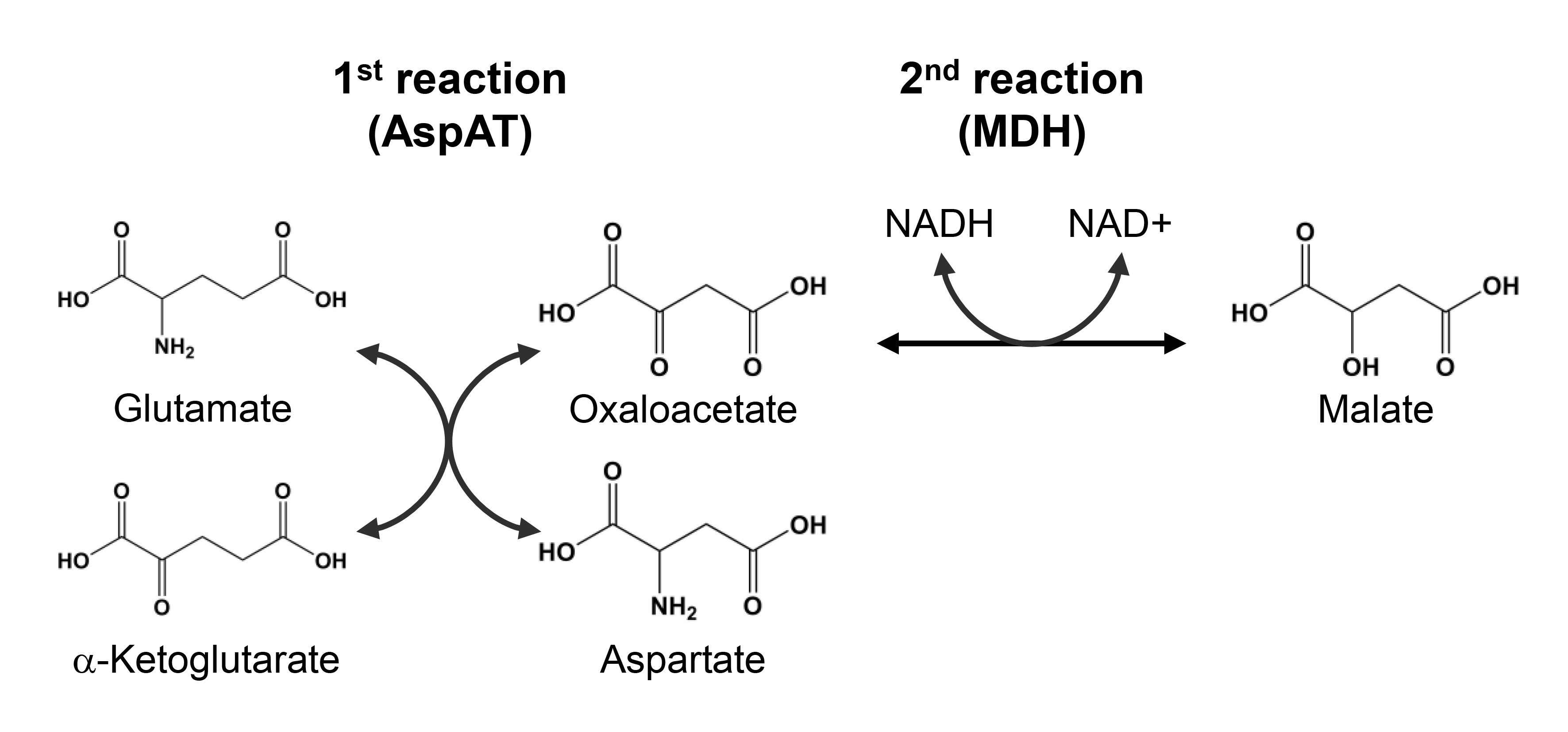

Supplement: S1 Fig — Coupled reaction is used to measure aminotransferase activity. At the 1st reaction and 2nd reaction, aspartate aminotransferase (AspAT) and malate dehydrogenase (MDH) are used respectively, and the decrease amount of NADH is monitored at 340nm. (TIF) [file pone.0158402.s001.tif]

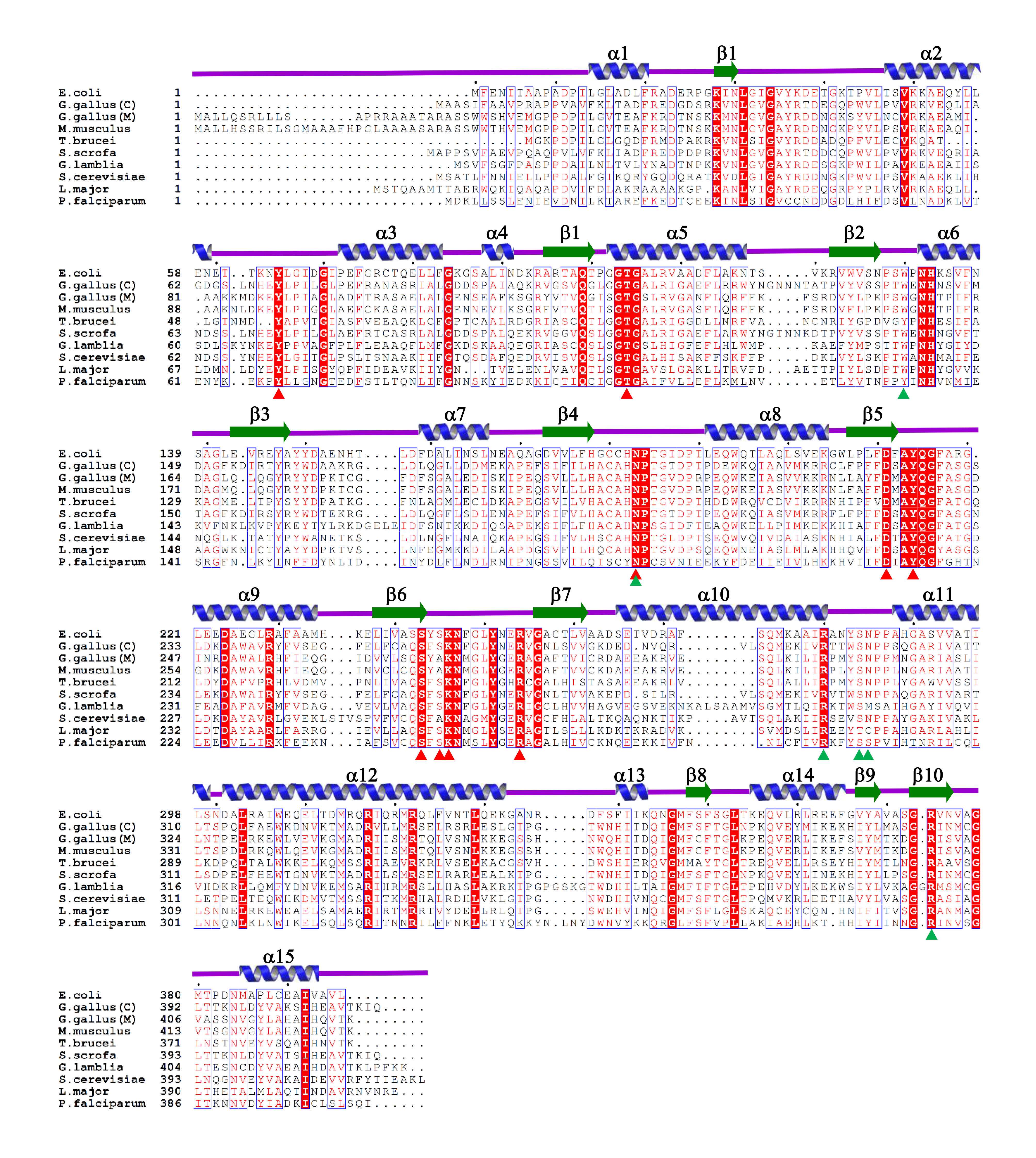

Supplement: S2 Fig — Amino acid sequences of 10 representative AspAT enzymes from subgroup Ia are aligned using Clustal Omega and ESPript software. The secondary structure elements are shown based on the structure of AspAT from E. coli. Residues involved in the binding of the PLP cofactor and the glutamate substrate are indicated by red- and green-colored triangles. (TIF) [file pone.0158402.s002.tif]

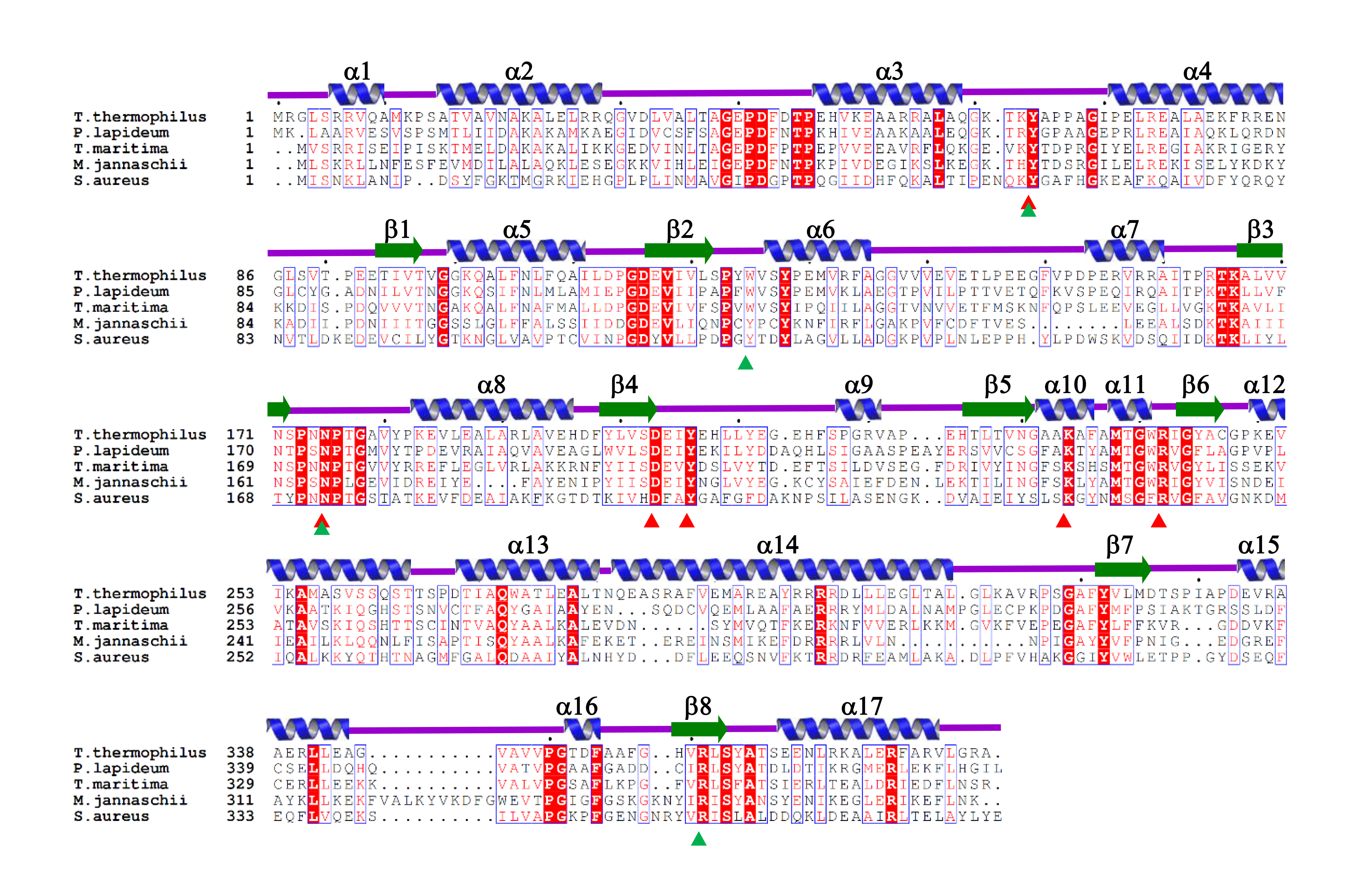

Supplement: S3 Fig — Amino acid sequences of 5 representative AspAT enzymes from subgroup Ib are aligned using Clustal Omega and ESPript software. The secondary structure elements are shown based on the structure of AspAT from Thermus thermophilus. Residues involved in the binding of the PLP cofactor and the glutamate substrate are indicated by red- and green-colored triangles. (TIF) [file pone.0158402.s003.tif]

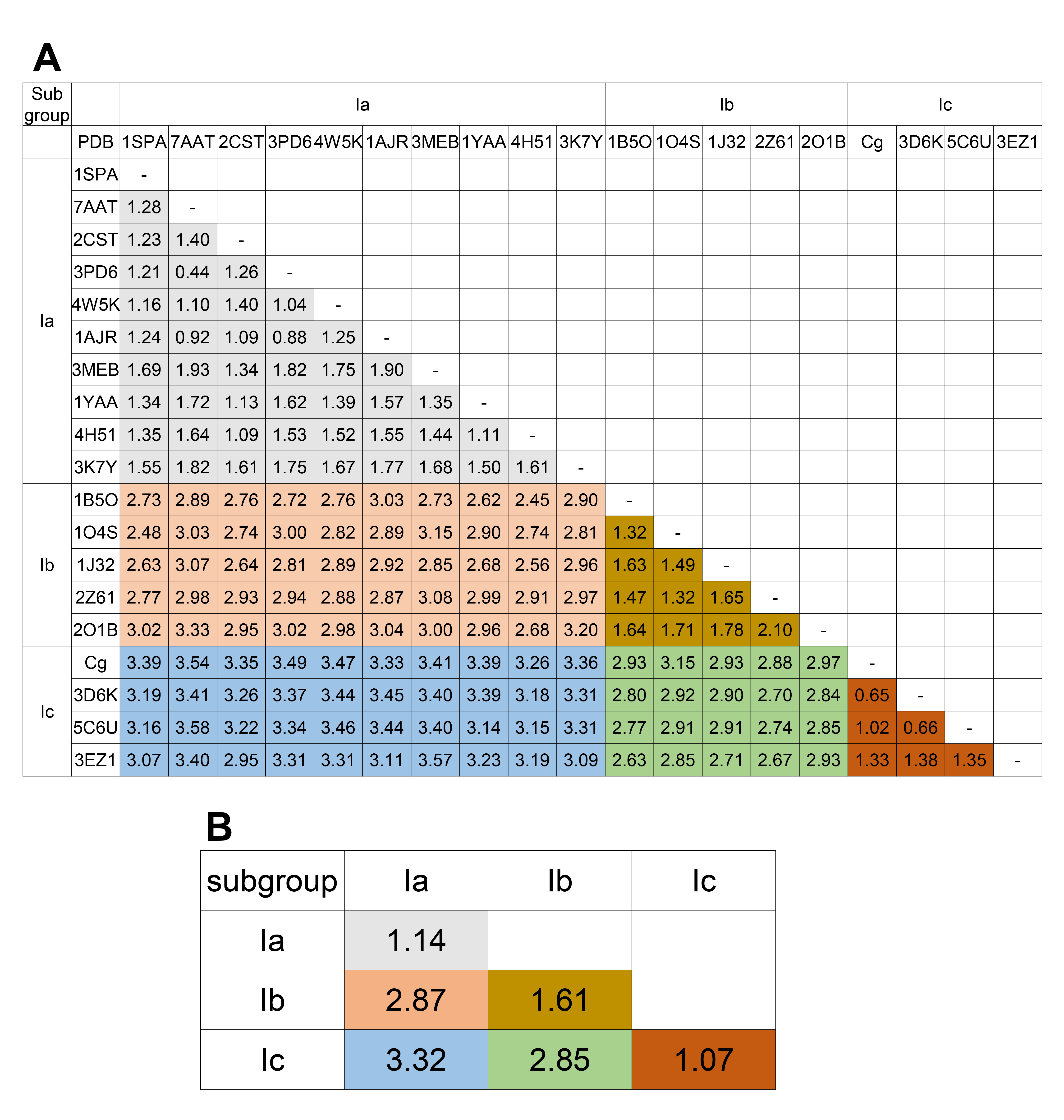

Supplement: S1 Table — (A) RMSD of reported AspAT structures were analyzed. PDB code 1SPA; Escherichia coli, 7AAT; Gallus gallus (cytosolic), 2CST; Gallus gallus (mitochondrial), 3PD6; Mus musculus, 4W5K; Trypanosoma brucei, 1AJR; Sus scrofa, 3MEB; Gaiardia lamblia, 1YAA; Saccharomyces cerevisiae, 4H51; Leishmania major, 3K7Y; Plasmodium falciparum, 1B5O; Thermus thermophilus, 1O4S; Thermotoga maritima, 1J32; Phormidium lapideum, 2Z61; Methanocaldococcus jannaschii, 2O1B; Staphylococcus aureus, Cg; Corynebacterium glutamicum, 3D6K; Corynebacterium diphtheriae, 5C6U; Mycobacterium tuberculosis, 3EZ1; Deinococcus geothermalis. (B) Average RMSD values between AspAT structures of 3 different subgroups. (TIF) [file pone.0158402.s004.tif]
